# Invisible Care: Nurses’ Lived Experiences of How the Practice Environment Shapes Nurse–Patient Relationships

**DOI:** 10.1155/jonm/1787140

**Published:** 2026-06-28

**Authors:** Miriam Pereira-Sánchez, Amparo Zaragoza-Salcedo, Mónica Vázquez-Calatayud, Maribel Saracíbar-Razquin

**Affiliations:** ^1^ Faculty of Nursing, University of Navarra, C/ Irunlarrea 1, 31008, Pamplona, Navarra, Spain, unav.edu; ^2^ Teaching Practice Unit, Faculty of Nursing, University of Navarra, Pamplona, Navarra, Spain, unav.edu; ^3^ Innovation for a Person-Centred Care Research Group, Faculty of Nursing, University of Navarra, Pamplona, Navarra, Spain, unav.edu; ^4^ Navarra’s Health Research Institute (IdisNA), Pamplona, Navarra, Spain, idisna.es; ^5^ Nursing Care for Adult Patients Department, Faculty of Nursing, University of Navarra, Pamplona, Navarra, Spain, unav.edu; ^6^ Clínica Universidad de Navarra, Av. de Pío XII 36, 31008, Pamplona, Navarra, Spain, cun.es; ^7^ Area of Nursing Professional Development and Research, Clinica Universidad de Navarra, Pamplona, Navarra, Spain, cun.es

**Keywords:** nurse-to-patient relationships, nursing leadership, nursing workload, organizational culture, patient-centered care, practice environment

## Abstract

**Aim:**

To explore how the nursing practice environment shapes nurse–patient relationships, identifying key organizational challenges and opportunities for nursing management and policy.

**Design:**

A qualitative hermeneutic phenomenological design was used to explore the lived experiences of nurses in a hospital setting.

**Methods:**

Data were collected through in‐depth, face‐to‐face interviews and analyzed using Van Manen’s hermeneutic phenomenological method. Data saturation was reached after 20 interviews.

**Results:**

The findings reveal that nurse–patient relationships are fundamentally shaped by organizational and structural conditions that systematically constrain the relational dimension of nursing practice. Nurses experienced a persistent tension between their professional commitment to person‐centered care and an organizational context that prioritizes task completion over relational engagement. Five themes were identified: (1) organizational culture fails to adequately recognize the relational essence of nursing; (2) environmental factors constrain team dynamics and nurse–patient relationships; (3) ambiguity in patient assignment undermines continuity of care; (4) limited time availability hinders meaningful patient engagement; and (5) high nurse–patient ratios erode the conditions for relational care.

**Conclusion:**

Meaningful nurse–patient relationships are structurally shaped by the practice environment. Strengthening nursing leadership, promoting professional autonomy, and redesigning organizational policies are essential to sustaining person‐centered care and improving patient outcomes.

## 1. Introduction

The professional nursing practice environment (PNPE)—also referred to in the literature as the nurse work environment (NWE)—is a critical determinant in healthcare organizations and a foundational element of professional nursing [1–3]. Defined as the “organizational characteristics of a work setting that facilitate or constrain professional nursing practice” [4], it encompasses not only physical conditions but also ethical principles, leadership, organizational support, and opportunities for continuous professional development that enable nurses to practice to their full potential [1, 2, 5–7]. The American Nurses Association (ANA) [8] also emphasizes the importance of an ethical and safe work environment that fosters quality care and nurse well‐being. Furthermore, a concept analysis of professionalism in nursing underscores that the professional environment comprises multidimensional attributes—knowledge, attitudes, and behaviors—crucial for effective clinical practice [9].

In recent years, multiple factors have challenged nursing practice environments: scientific and technological advances, economic crises, evolving societal demands, increased life expectancy, rising prevalence of chronic conditions, and pandemics such as COVID‐19 [10]. These developments have significantly impacted healthcare systems and the nursing profession. Simultaneously, nursing has increasingly embraced a person‐centered care model [2, 6, 11, 12], placing the nurse–patient relationship at the core of its practice. Within this framework, nursing emphasizes a caring relationship grounded in the individual’s health experience [13–15].

To support this paradigm shift, nursing practice environments must foster conditions that allow nurses to prioritize the person, contributing to both their professional development and the quality of care delivered. Consistent with this, the 2023 Annual Impact Report by the ANA highlights the progress made in improving practice environments, stressing the need to advance the profession through inclusive, equitable, and technologically forward workplaces [16].

Understanding how the nursing practice environment specifically shapes nurse–patient relationships is, therefore, essential to advancing person‐centered care and informing nursing management practice.

## 2. Background

The growing complexity of healthcare environments and their impact on nursing have prompted extensive research into the factors that influence both nursing practice and the work environment. Early studies focused on the excellence of nursing practice and the quality of work environments, particularly in Magnet hospitals [17–21]. These studies identified key factors associated with high‐quality nursing practice and explored their relationship with organizational performance [22–24]. As research evolved, particularly influenced by the person‐centered care approach, the focus shifted toward examining the quality of patient care, including issues such as the omission of care [25–27]. Additionally, several studies explored the relationship between the nursing practice environment and outcomes for both nurses and patients [28–31]. Challenges, such as the focus on routines rather than individualized care, have been identified as barriers to effective interpersonal relationships in nursing practice [32–35]. However, to date, no studies have directly and systematically examined how the PNPE shapes interpersonal nurse–patient relationships from nurses’ lived experience. This constitutes a significant gap in the literature, given that these relationships are central to person‐centered care and directly mediated by the conditions of the practice environment. Nurses, as the primary actors in these interactions, offer a unique perspective for exploring this phenomenon. Their insights can inform policies and professional practice models, promote optimal practice environments, and foster stronger, more effective nurse–patient relationships.

## 3. Methods

### 3.1. Aim

To understand, from nurses’ experience, the significance of the PNPE within the context of the nurse–patient relationship.

### 3.2. Study Design

A qualitative study using a hermeneutic phenomenological approach was conducted, based on the phenomenology of practice method proposed by van Manen [36]. This methodology seeks to reflect on professionals’ day‐to‐day practice. The research methods in this approach are both descriptive and interpretative, providing a description of the event or lived experience and interpreting its meaning.

### 3.3. Theoretical Framework

This study is grounded in the “Model of interpersonal relationship between the nurse and the person/family cared for” [3]. This model served as a guiding framework, helping researchers explore the phenomenon under study. It emphasizes person‐centered nursing care, fostering the development of nursing knowledge, advancing the nursing profession, and promoting educational innovation [37–43].

In this model, nursing is conceptualized as the interpersonal relationship that nurses establish with the person/family cared for [3]. The focus is on understanding the health experiences of these individuals and families, helping them find meaning in these experiences and in their lives. According to Saracíbar‐Razquin, nursing involves supporting individuals and families in enhancing their potential and ensuring their well‐being.

The model also expands the concept of “environment,” understanding it not only as the physical space but as a dynamic and open reality, constantly interacting with broader contexts. This environment encompasses the organization’s ideology, culture, governance structure, objectives, and the professional nursing practice model. The nurse, too, is an integral part of this environment. The interpersonal relationship between nurses and the person/family is shaped within this PNPE, which, depending on its characteristics, can either positively or negatively influence the quality of care or even hinder it.

### 3.4. Setting and Participants

The sample consisted of nurses from a private teaching hospital in northern Spain, an internationally recognized center that integrates patient care, research, and teaching into excellent clinical practice, prioritizing patients and their needs. The inpatient units included in the study were: Oncology, General Surgery, Cardiology, Internal Medicine, Digestive, and Gynecology. Inclusion criteria encompassed nurses working in adult inpatient units in the selected areas, with voluntary participation and completion of a consent form. Exclusion criteria included nurses in training, advanced practice nurses, nursing supervisors, and those working exclusively on the night shift. This sample was selected through purposeful sampling, ensuring that participants had significant experience with the phenomenon under investigation [44]. Data saturation was reached when no new interpretations emerged [45]. Sufficient experiential material was available to offer in‐depth descriptions and interpretations [36]. The first author personally provided brief information about the study to each potential nurse participant. Nurses expressed their willingness to participate by contacting the researcher via telephone. During this call, the researcher addressed any questions or concerns and scheduled the interview’s date, time, and location. It is important to note that this phone call was the only interaction between the researcher and the nurses prior to the interviews.

### 3.5. Data Collection

A single face‐to‐face conversational interview was conducted with each nurse by the first author between June 2019 and May 2021. Data collection was paused from March to August 2020 due to the global coronavirus disease pandemic. The average duration of each interview was 33 min, and the interviews were held in one of the staff rooms within the inpatient units. All interviews were recorded and transcribed verbatim. The interviews began with an open question: Could you tell me how your last shift has gone? This question allowed nurses to reflect on their personal experience in the practice environment. Nurses were not directly asked about issues related to the nursing practice environment; however, this theme emerged organically during the conversation. In instances where nurses had difficulty elaborating on these topics, a thematic guide was provided to help them to focus on the study objectives (Table 1).

**TABLE 1:** Interview guide.

*Beginning of the interview* To begin, could you tell me how your last shift went? Feel free to share whatever you find interesting.

*Some guiding questions to keep the conversation flowing* (a) What were your interactions with your patients for? Is this typical? Is there anything concerning you in this regard? (b) Do you usually see all your patients during your shift? What care did you provide them? (c) How often do you enter their rooms? For what reasons? What is your interaction like during your time in their rooms? What makes you feel good in those instances? Is there anything worrying you?

*Additional prompts to expand the conversation* (a) Could you elaborate more on that topic? (b) Could you explain this in a bit more detail? (c) What do you mean by this? (d) Can you provide a recent example of this? (e) Let me see if I’ve understood correctly, could you give me a recent example… (f) I’m not sure if I’ve understood what you mean by this… (g) Could you tell me more about this? (h) In what instances? How did it happen? (i) Would you like to add anything else on this topic?

*End of the interview* As we conclude, among everything we’ve discussed, what would you highlight as the most important? Is there anything that concerns you particularly? Is there anything you’d like to mention that hasn’t come up yet, but you find interesting?

### 3.6. Data Analysis

Data analysis followed the thematic reflection process inherent to Van Manen’s phenomenology of practice [36]. This approach sought to uncover the internal meaning structures or “essences” of the experiential narrative—elements without which the experience would not be the same. In identifying themes, the two levels of thematic reflection proposed by van Manen were applied. First, a macrothematic reflection or holistic approach was conducted, including a narrative summary of each nurse’s experience. Subsequently, a microthematic reflection was carried out, involving both a selective approach and a detailed reading approach. Throughout this process, the researchers applied *epoché reduction*, consciously reflecting on and setting aside their preconceptions. The interpretative phenomenological approach assumes a co‐creation of data between the researcher and participant, where both jointly generate and interpret data through their interactive process. Additionally, the approach draws on the hermeneutic circle, a circular process of interpretation in which the understanding of individual parts and the whole continuously influence one another [46]. Data analysis was conducted by three members of the research team in the following sequence: (1) the first author analyzed the interviews individually; (2) the first and last authors reviewed the interviews together; and (3) the first, last, and second authors conducted a comprehensive joint analysis as part of the broader research team. QSR International’s NVivo 10 qualitative data analysis software was used to organize the data. This analysis process facilitated the identification of the themes that encompassed the nurses’ experience (Table 2).

**TABLE 2:** Examples of the analysis process.

Wholistic reading approach	Selective approach	Detailed approach	Theme
“Certain circumstances in the practice environment are affecting the nursing team’s atmosphere and the relationship nurses have with patients.”	“The workload now is nothing like it used to be, [the nurses] feel frustrated, unable to provide patients with adequate care… People are tired, it is tense, and among us, it is not a very appropriate environment… sometimes one gets angry, doesn’t talk to the other… and then when you enter the patient’s room, you might not enter with the greatest joy.”	“The high workload creates significant frustration due to the inability to provide patients with adequate quality of care”	Environmental factors affect the nursing team’s atmosphere and, consequently, their relationships with patients.
“The lack of continuity in patient assignment prevents me from getting to know them.”	“In continuity with the patient, the patient knowing you, that’s where you risk a lot in terms of your relationship with them.”	“Due to the lack of continuity with patients, I’m unable to establish the same depth of relationship with them”	Experiencing ambiguity in patient assignment: between continuity and change
“Having a high nurse‐to‐patient ratio doesn’t allow me to provide quality care to patients.”	“There were two nurses… then suddenly they decided to open another bed… our nurse‐patient ratio was already exceeded, right? So that means that you take one, and your colleague takes another… if there were more people… then you would have time to provide good care and have a relationship with the patient.”	“If there were more nurses, I would have time to engage in conversations with patients”	The impact of high nurse–patient ratios on patient relationships

### 3.7. Ethical Considerations

The study was approved by the Research Ethics Committee of the Universidad de Navarra (CODE 2018/043) and the hospital’s management team. It adhered to the principles outlined in the Declaration of Helsinki [47]. Participants received both oral and written information, with a focus on emphasizing their voluntary participation, confidentiality, and data anonymity. Additionally, they were informed that their participation would not impact their current work at the hospital, either positively or negatively. An informed consent form was signed by each nurse before beginning the interview. The first author encoded participants’ identities, ensuring that participants and no other researchers had access to identifying information.

## 4. Results

A total of 20 nurses participated in this study. The participants’ ages ranged from 23 to 47 years, with a mean age of 33.75 years. All participants were women. Regarding educational background, eight nurses held master’s degrees, while twelve had bachelor’s degrees. All the nurses, except one, had specialized training. Professional experience ranged from 1.5 to 24 years, with a mean of 10.8 years.

The nurses’ experience was grouped into five main themes: (1) The organization’s culture does not adequately recognize the essence of nursing; (2) environmental factors affect the nursing team’s atmosphere and, consequently, their relationships with patients; (3) experiencing ambiguity in patient assignment: between continuity and change; (4) experiencing a lack of time to personally engage with patients; and (5) the impact of high nurse–patient ratios on patient relationships. These themes and their definitions are presented in Table 3.

**TABLE 3:** Main themes of the participating nurses.

Theme 1	The organization’s culture does not adequately recognize the essence of nursing *Nurses state that both unit management and collaborative work with the medical team highlight that the organization still does not recognize the professional responsibilities of nursing*

Theme 2	Environmental factors affect the nursing team’s atmosphere and, consequently, their relationships with patients *The nurses report that factors such as workload and the clinical condition of patients particularly affect the nursing team’s environment. They explain that this impacts the relationship they build with patients*

Theme 3	Experiencing ambiguity in patient assignment: between continuity and change *The nurses state that they usually rotate the patients assigned to them weekly to maintain their personal well-being. However, they also express that this negatively impacts their personal knowledge of the patients and the trust relationship they establish*

Theme 4	Experiencing a lack of time to personally engage with patients *The nurses explain that the complexity of the patients’ clinical situations, along with the high level of activity this entails in the unit, forces them to focus on the physical aspect of care and on diagnostic and/or therapeutic procedural activities, to the detriment of the nurse–patient interpersonal relationship*

Theme 5	The impact of high nurse–patient ratios on patient relationships *The nurses express that the high patient workload they have, due to the shortage of nurses, leads them to prioritize “getting the work done” and focus on caring for the most complex patients*

*Theme 1: The organization’s culture does not adequately recognize the essence of nursing*. Nurses perceived that organizational culture fails to acknowledge the full scope of nursing competencies, prioritizing task‐oriented and medically driven activities over the relational dimensions of care. This manifested in two interconnected ways: the invisibility of relational nursing work within documentation and accountability systems, and the undervaluation of nurses’ professional expertise in interdisciplinary interactions. Below are some experiences shared by nurses that illustrate this perception:

When I changed the IV line… It is like a minimum… then you must document it, and it is like work that you must account for… there is work that is not visible… that you will not document… It is that other part of nursing… that we often don′t give importance to because it seems like the symptom, the symptom… And what about the patient′s perception and how it is affecting them psychologically and how they are coping? (Participant, E18, transcript)

Physicians’ rounds frequently disrupted nurses’ workflow, preventing focused patient‐centered care and reinforcing a perception that nursing responsibilities were poorly understood by other professionals:

They start saying what they think a nurse does, and for them, it is dressing wounds, making coffee, administering medication, calling the resident all the time. Of course…, when you recount your reality, they say: really?… It strikes you because they have no idea about nursing work. (Participant, E1, transcript)

Another nurse elaborated on the disruption caused by physicians during rounds: “At the start of the eight o’clock rounds, doctors come to start wound dressings, to discharge patients… that already interrupts… delaying your work, and then you are rushing through everything… you can’t give the attention you would like…” (Participant, E11, transcript).

In addition to these interruptions, which affected both nurses’ efficiency and their sense of professional value, this situation further reinforced their feeling that their professional competencies are not being respected. Challenges with task delegation compounded this dynamic. Nurses frequently assumed responsibilities beyond their role because nursing assistants failed to respond to delegated tasks, increasing workload and reducing time available for patient interaction. One nurse explained:

The assistants… could do more work than they do… we′re tired of delegating tasks or telling them what they should do… in the end, you end up doing it yourself… that overloads you… You end up doing things that shouldn′t be your responsibility… for the patient′s sake… it overloads you… And that affects your relationship with them. (Participant, E12, transcript)

These experiences reveal that organizational culture renders the relational dimension of nursing structurally invisible, subordinating interpersonal care to task‐oriented priorities.

*Theme 2: Environmental factors affect the nursing team’s atmosphere and, consequently, their relationships with patients*. Excessive workload and clinical complexity of patients emerged as primary forces shaping the emotional climate of the nursing team, with direct consequences for nurse–patient interactions. Nurses described how sustained work pressure generated frustration and interpersonal tension among colleagues, which inevitably carried over into their encounters with patients:

The workload now is nothing like it used to be, [the nurses] feel frustrated, unable to provide patients with adequate care… People are tired, it is tense, and among us, it is not a very appropriate environment… sometimes one gets angry, doesn′t talk to the other… and then when you enter the patient′s room, you might not enter with the greatest joy. (Participant, E8, transcript)

In addition to the negative atmosphere, nurses observed that heavy workloads also disrupted communication and mutual support within the team, eroding the collegial conditions that sustain safe and attentive care:

People are a bit burnt out by the type of patients we have [here]… it has a big influence if in your environment you have a colleague on shift who says: oh, how are you doing? Hey, if you need anything, let me know. That also gives you a lot of peace in your work, and you think it is okay because of that and that. Another person might be complaining all the time, and you think: as if I would ask them for anything! (Participant, E14, transcript)

Additionally, nurses further identified insufficient leadership responsiveness as a compounding factor. The perception that nurse managers neither acknowledged nor addressed their workload concerns deepened feelings of professional undervaluation and frustration. One nurse described this frustration as follows:

If you′ve been giving a nurse a workload for five years that doesn′t correspond to their responsibility… if you demand that a nurse provide quality care but at the same time demand that she takes on more workload than she should to provide that care… Well, I understand that can be asked at a time when perhaps things are busier, and that is okay, but when you ask for a favor and ask for another and ask for another and ask for another, and it goes on for years, and you don′t listen and don′t give anything in return, and on top of that, the relationship is purely professional, well, the nurse feels completely undervalued and unheard. (Participant, E18, transcript)

Collectively, these experiences reveal that the work atmosphere operates as a mediating factor between organizational conditions and the quality of nurse–patient interactions—when the environment is emotionally depleted, relational care becomes a residual rather than a central element of practice.

*Theme 3: Experiencing ambiguity in patient assignment: between continuity and change*. Nurses expressed a dual perspective regarding patient assignment. On one hand, they acknowledged the need for rotating patient assignments to prevent burnout and promote psychological well‐being, particularly when caring for patients with complex clinical needs. Nurses highlighted the benefits of varying patient assignments, explaining that it allowed them to engage in different tasks and reduce the burden of repetitive care responsibilities. As one nurse explained:

There are chronic patients who do require a lot of care, and in the end, it is burdensome to be with the same patient all week doing the same things. So, if you change, if you change dressings, if you change patients, specialties, it really makes a difference. (Participant, E5, transcript)

On the other hand, despite the advantages of rotating assignments, nurses also acknowledged the importance of continuity in patient care, particularly in building personal relationships. They emphasized that the lack of continuity often prevents them from developing meaningful connections with patients, as they are frequently assigned to new patients they do not know. This discontinuity hampers the depth of the nurse–patient relationship, making it more difficult to establish effective communication and rapport. As one nurse described:

What prevents me from having a relationship with the patient or developing a deep relationship with them is not knowing them. I… don’t have much continuity with the patient because I arrive…, they introduce me to the patient, and it’s the first day I meet them, so you can′t build the same kind of relationship. (Participant, E14, transcript)

This ambivalence reflects a fundamental tension in nurses’ lived experience: the need for self‐protection through variety conflicts with the relational continuity that meaningful care requires.

*Theme 4: Experiencing a lack of time to personally engage with patients*. Nurses consistently identified time scarcity as a central barrier to meaningful nurse–patient relationships. The combination of clinical complexity and administrative demands left little room for the relational engagement they considered integral to their professional role. As one nurse explained:

There are times when, no matter how much you want to, it′s just not feasible… it′s not feasible because of the workload and the time constraints… what I mean is, there′s a lot of medication, for example, with transplant patients, you can′t imagine how many times we go into their rooms just to change medication, like antibiotics, painkillers, for nausea… while you are doing that or while you are at the computer filling out all the necessary paperwork, obviously, you are not with the patient. (Participant, E17, transcript)

Another nurse elaborated on the challenge of finding time for meaningful engagement:

Sometimes, we go in too quickly… I would like to… be able to spend more time with patients and take care of them more… to listen more, that′s what I would love, but the workload of the unit makes it impossible… (Participant, E2, transcript)

Despite their strong desire to provide comprehensive care, this time pressure led nurses to consciously limit relational depth as a coping strategy, accepting superficiality as an unavoidable feature of their practice. As one nurse expressed:

I see and notice that during the busiest times, my relationship is much more superficial, that is a reality, that is how it is… because I don′t have time, I really don′t have time. So, I say, it′s better not to have a deeper relationship because I won′t achieve it, so I prefer to just go over things quickly, plan a bit with that patient, not complicate my shift. (Participant, E2, transcript)

These experiences point to a lived paradox: nurses are physically present with patients yet relationally absent, their time consumed by clinical and administrative demands that preclude the very engagement that defines person‐centered care.

*Theme 5: The impact of high nurse–patient ratios on patient relationships*. Staffing shortages and high nurse–patient ratios were experienced as structural constraints that directly limited nurses’ relational availability. When patient loads exceeded safe thresholds, the time and attention required for meaningful interaction became systematically unavailable. As one nurse described:

There were two nurses… then suddenly they decided to open another bed… our nurse‐patient ratio was already exceeded, right? So that means that you take one, and your colleague takes another… if there were more people… then you would have time to provide good care and have a relationship with the patient.” (E14) (Participant, E18, transcript)

When the nurse–patient ratio was too high, nurses faced increased workloads and less time for personal interactions with patients. The impact was particularly acute with clinically complex patients, whose care demands left no margin for the individualized attention nurses sought to provide:

I would like to spend more time with the patients and for the clinical complexity of the patients we have hospitalized to be valued. I don’t know, to have a better understanding of each patient’s burden, whether they need a lot of care or less… (Participant, E19, transcript)

In addition, nurses noted that high ratios also redirected nurses’ focus toward task completion at the expense of relational care, contributing to professional frustration and burnout:

There are shifts when… we’ve had around 10 patients… that affects the patient relationship a lot because before, we could provide more individualized care… talk with the patient about their life, their concerns… now, due to the lack of time with more patients… you only have enough time to administer medication. (Participant, E8, transcript).

Similar to the challenges discussed in Theme 2, nurses further noted that managers were often unresponsive to staffing pressures, advocating for proactive measures such as anticipating workload fluctuations and providing timely reinforcements. As one nurse explained:

So, the management? Well, that′s what I′m saying. I understand it is difficult, but well, depending on the type of patient, if reinforcements are needed, if you see that things are getting complicated… I think reinforcements should be considered, you know? Anticipating or seeing if someone is more complicated, I mean asking, how are things going? How are the patients, are they manageable, not manageable, are they with a caregiver, without a caregiver? It is true that often… I don′t know if the supervisor focuses on that and so on, but practically speaking, there are times when support is lacking during shifts. (Participant, E19, transcript)

Underlying all these accounts is the lived experience of structural scarcity—a condition in which the organizational decision to increase patient load is experienced by nurses as a direct erosion of their capacity to care relationally.

## 5. Discussion

### 5.1. Main Findings

To our knowledge, this is the first qualitative study in our country to explore how the nursing practice environment influences nurse–patient relationships, addressing a significant gap in international literature. The findings reveal a consistent pattern across themes: organizational and structural conditions systematically constrain nurses’ capacity for relational engagement, positioning the practice environment as a fundamental determinant of person‐centered care.

This study also highlights the value of a phenomenological–hermeneutic approach in capturing nurses’ lived experiences, offering deeper insight into their practice. Grounded in the “Model of Interpersonal Relationship between the Nurse and the Person/Family Cared For” [3], our research underscores that person‐centered care can only be achieved in a supportive practice environment. This conceptual framework guided both the data collection and interpretation processes, ensuring that our findings contribute to a deeper understanding of how hospital environments shape nurse–patient relationships. Below, we discuss the most novel contributions of this study, which not only complement but also extend previous research on this topic.

The lack of recognition for the essence of nursing and nurses’ professional responsibilities within organizational culture emerges as a critical issue in our study. Nurses reported that medical and diagnostic tasks often overshadow core nursing duties, limiting their ability to address patients’ broader needs. Time constraints systematically prevent nurses from building meaningful relationships with patients, even when they recognize the importance of these connections. This misalignment between professional responsibilities and institutional expectations contributes to the devaluation of nursing roles and fosters demotivation. Furthermore, nurses often take on non‐nursing tasks to fill gaps left by other healthcare professionals, which increases their workload and diminishes their autonomy. These findings align with those of Kelly et al. [48], who link a lack of professional recognition to higher levels of emotional exhaustion, while Pursio et al. [49] highlight the positive impact of a supportive work environment on job satisfaction and autonomy. However, our study adds a new perspective by emphasizing how the organizational culture itself directly shapes the quality of nurse–patient relationships, an aspect that has been underexplored in previous research. This points to the need for institutional changes that make the relational work of nursing organizationally visible and valued.

Another significant finding is the frequent interruptions nurses experience during their workflow, particularly from physicians. These disruptions force nurses to change their workflow and reinforce a perception that their professional responsibilities were poorly understood, consistent with previous studies linking interruptions to reduced care quality and increased occupational stress [50, 51]. This reflects a broader deficit in interdisciplinary collaboration, as Reeves et al. [52] associate weak collaboration with communication breakdowns and diminished respect between professionals. While Verspuy and Van Bogaert [53] underscore nurses’ key role in interdisciplinary teams due to their continuous patient contact, our findings reveal that this role is not consistently acknowledged in clinical decision‐making. Strengthening nursing leadership within healthcare teams is therefore essential to promoting a genuinely collaborative culture [2], and programs such as *Leadership Saves Lives* have shown positive effects on relational coordination and team cohesion [54].

To address these challenges, Nursing Professional Practice Models (NPPMs) offer a structured framework for clarifying nursing roles, enhancing professional recognition, and reinforcing person‐centered care [43, 55, 56]. However, their effectiveness depends on ongoing integration into daily clinical practice and systematic evaluation over time.

Environmental factors also play a critical role in shaping team dynamics and, by extension, nurse–patient relationships. Excessive workloads and stress disrupt team cohesion, negatively impacting both interpersonal relationships and patient care quality. Prior studies confirm that high workloads harm nurse well‐being and patient outcomes [57, 58]. However, we argue that the tension within nursing teams deserves more attention, as it constitutes a significant barrier to effective communication and collaboration. A cohesive, supportive team environment is essential for ensuring high‐quality care and the well‐being of nurses. When nurses work in a positive environment with strong team relationships, they are more likely to extend this positivity to their interactions with patients, ultimately improving care quality [59]. A well‐supported team environment is therefore a prerequisite for meaningful nurse–patient encounters, not merely a staff welfare concern.

Leadership from nursing managers is another critical factor. Nurses in our study expressed frustration with managers who failed to address their concerns, leading to feelings of undervaluation and stress. Strong leadership is essential for creating a positive work environment, as demonstrated by Cummings et al. [60] and Wong et al. [61], who associate effective leadership with reduced nurse stress and enhanced job satisfaction. Transformational leadership, which inspires and supports teams, has been shown to improve patient outcomes and staff retention [16]. Moreover, good leadership can alleviate environmental stressors and boost team morale [62, 63]. Our study reinforces the need for nurse managers to prioritize open communication, conflict resolution, and recognition of nursing contributions, alongside the implementation of support strategies such as wellness programs and workload management initiatives.

The ambiguity surrounding patient assignments also emerged as a key issue, particularly the tension between rotation (to prevent burnout) and continuity (to foster strong relationships). While consistent assignments can enhance patient trust and care outcomes [64], staff shortages and emotional strain often challenge this stability. Our study underscores the importance of a structured approach that balances rotation with continuity, helping to maintain relationship‐based care while mitigating burnout [65, 66]. Improved communication, psychological support, and individualized care planning are essential strategies to balance rotation with continuity.

Our study also highlights the detrimental impact of high nurse‐to‐patient ratios, which exacerbate excessive workloads and limit meaningful patient interactions. Staff shortages and increasing patient complexity force nurses to prioritize urgent tasks, often at the expense of essential care. Previous studies [67, 68] confirm the negative effects of high ratios on care quality and nurse–patient engagement. Nurses in our study expressed frustration with the lack of managerial support in advocating for better staffing policies. This points to the crucial role of leadership in addressing workload challenges and preventing burnout [60, 63]. Effective leadership at all levels is essential in driving staffing reforms and ensuring adequate resources for nurses to provide optimal care. Policymakers and healthcare institutions must implement strategies to optimize staffing levels, streamline workflows, and enhance managerial engagement. Internationally, countries such as the UK, Ireland, France, and Germany have introduced staffing regulations to improve nurse workloads and care quality [69]. However, in Spain, efforts remain inconsistent, with regional disparities in staffing standards [70]. In a recent press release, the General Council of Nursing [71] in Spain denounced this situation, emphasizing the urgent need to implement strategies to optimize staffing levels and improve workload management. Addressing high nurse‐to‐patient ratios requires coordinated policy reform at both institutional and national levels.

These findings collectively point to the practice environment as a structural determinant of nurse–patient relationships—one that cannot be addressed through individual interventions alone but requires systemic organizational and policy responses that place relational care at the center of nursing management.

### 5.2. Study Limitations

This study has several limitations. First, as a phenomenological study, the findings are not generalizable to all nursing practice settings, as qualitative research methods do not allow for population representativeness. Therefore, further multicenter research with a larger nurse sample is needed to enhance the external validity of the findings. Second, the researchers, acting as the primary data collection tool, may introduce interviewer bias. To ensure credibility and mitigate this risk, we engaged in self‐reflection to avoid the influence of a priori assumptions and employed triangulation for data analysis. Third, the interviews were conducted during the COVID‐19 pandemic, a period marked by strict infection control measures and increased workloads for healthcare professionals, which may have influenced the study’s outcomes. Finally, while Van Manen’s phenomenological approach guided the study, the analysis did not explicitly examine participants’ experiences through the lens of the lifeworld existentials. Future studies using this framework may provide a richer understanding of how nurses experience time, space, embodiment, and relationships within clinical environments, thereby informing more targeted recommendations for practice, management, education, and health policy.

### 5.3. Implications for Practice and Research

The findings of this study provide valuable insights into the challenges faced by nursing staff in clinical environments and highlight critical areas for management intervention to improve care quality and foster stronger nurse–patient relationships.

Firstly, there is a pressing need for healthcare organizations to explicitly recognize and integrate the nursing role within their organizational culture. Valuing nursing contributions at all levels enhances professional identity, promotes interdisciplinary collaboration, and strengthens patient‐centered care delivery.

Reducing administrative burdens through the strategic implementation of efficient digital solutions must be prioritized to optimize nurses’ time for direct patient care and relationship‐building activities.

Effective leadership from nurse managers emerges as a pivotal factor in creating and sustaining positive work environments. Leadership development programs, emotional support initiatives, and team‐building strategies are essential tools to foster professional autonomy, enhance staff motivation, and reduce turnover rates. Establishing open channels for interdisciplinary communication can further strengthen care coordination and team cohesion. Nursing management education should explicitly recognize the nurse–patient relationship as a fundamental component of high‐quality care. Consequently, a central objective of leadership and management development should be to equip nurse managers with the knowledge and skills required to create, protect, and sustain organizational conditions that enable meaningful nurse–patient relationships. This includes staffing decisions, workflow design, workload allocation, and the promotion of cultures that value relational care alongside technical and operational outcomes.

From a management research perspective, further exploration is needed into the organizational factors influencing nurse–patient relationships. Future studies should also seek to quantify the time required to establish, maintain, and sustain therapeutic nurse–patient relationships across different clinical settings. Such evidence could support the development of more comprehensive workload measurement systems and staffing models that account not only for procedural and technical tasks but also for the relational work that is fundamental to nursing practice. Additionally, evaluating the impact of innovative administrative streamlining technologies, the adoption of NPPMs, and the influence of leadership development programs on team dynamics and care outcomes will offer valuable guidance for best management practices.

By addressing recognition, workload, leadership strategies that create space for essential nurse–patient relationship building, and collaboration challenges, nursing management can directly contribute to both improved patient outcomes and the sustainability of the nursing workforce.

## 6. Conclusion

This study reveals critical organizational challenges that impede the development of meaningful nurse–patient relationships. Insufficient recognition of the nursing role within institutional culture, administrative overload, ineffective leadership, and frequent workflow interruptions undermine nurses’ ability to provide person‐centered care. These barriers not only compromise care quality but also significantly impact nurses’ emotional well‐being and team cohesion.

The findings highlight the urgent need for healthcare organizations to foster supportive practice environments that recognize the fundamental contributions of nursing, reduce administrative burdens, and promote sufficient time for meaningful patient engagement. Strengthening nursing leadership and enhancing interdisciplinary collaboration are strategic priorities for improving both patient outcomes and staff satisfaction.

From a research perspective, this study underscores the necessity of further investigating the influence of environmental factors on nurse–patient interactions, evaluating technological solutions to administrative overload, and assessing the implementation of NPPMs to enhance care quality. Future research should aim to integrate these elements to create more sustainable, effective, and resilient nursing practice environments.

## Author Contributions

Miriam Pereira‐Sánchez: conceptualization, methodology, formal analysis, investigation, writing–original draft, writing–review and editing, visualization, and funding acquisition.

Amparo Zaragoza‐Salcedo: conceptualization, methodology, formal analysis, investigation, writing–review and editing, visualization.

Mónica Vázquez‐Calatayud: writing–review and editing, visualization, and funding acquisition.

Maribel Saracíbar‐Razquin: conceptualization, methodology, formal analysis, investigation, writing–original draft, and visualization.

## Funding

The research leading to these results has received funding from “la Caixa” Banking Foundation and by the Research Plan of the Universidad de Navarra (Plan de Investigación de la Universidad de Navarra, PIUNA) (N° 2018‐25).

## Ethics Statement

The study was approved by the Research Ethics Committee of the Universidad de Navarra (CODE 2018/043).

## Conflicts of Interest

The authors declare no conflicts of interest.

## Data Availability

The data that support the findings of this study are available from the corresponding author upon reasonable request.
